# High Dietary Fat Intake during Lactation Promotes the Development of Social Stress-Induced Obesity in the Offspring of Mice

**DOI:** 10.3390/nu7075257

**Published:** 2015-07-17

**Authors:** Tsuyoshi Tsuduki, Kazushi Yamamoto, Shuang E, Yu Hatakeyama, Yu Sakamoto

**Affiliations:** Laboratory of Food and Biomolecular Science, Graduate School of Agriculture, Tohoku University, Sendai 981-8555, Japan; E-Mails: kazushi.yamamoto.t1@dc.tohoku.ac.jp (K.Y.); eshuang0@yahoo.co.jp (S.E.); yu.hatakeyama.q5@dc.tohoku.ac.jp (Y.H.); szsnyu10@dc.tohoku.ac.jp (Y.S.)

**Keywords:** lactation, maternal nutrition, obesity, offspring, stress

## Abstract

This study examined how a maternal high-fat diet (HD) during lactation and exposure of offspring to isolation stress influence the susceptibility of offspring to the development of obesity. C57BL/6J mice were fed a commercial diet (CD) during pregnancy and a CD or HD during lactation. Male offspring were weaned at three weeks of age, fed a CD until seven weeks of age, and fed a CD or HD until 11 weeks of age. Offspring were housed alone (isolation stress) or at six per cage (ordinary circumstances). Thus, offspring were assigned to one of eight groups: dams fed a CD or HD during lactation and offspring fed a CD or HD and housed under ordinary circumstances or isolation stress. Serum corticosterone level was significantly elevated by isolation stress. High-fat feeding of offspring reduced their serum corticosterone level, which was significantly elevated by a maternal HD. A maternal HD and isolation stress had combined effects in elevating the serum corticosterone level. These findings suggest that a maternal HD during lactation enhances the stress sensitivity of offspring. White adipose tissue weights were significantly increased by a maternal HD and isolation stress and by their combination. In addition, significant adipocyte hypertrophy was induced by a maternal HD and isolation stress and exacerbated by their combination. Thus, a maternal HD and isolation stress promote visceral fat accumulation and adipocyte hypertrophy, accelerating the progression of obesity through their combined effects. The mechanism may involve enhanced fatty acid synthesis and lipid influx from blood into adipose tissue. These findings demonstrate that a maternal HD during lactation may increase the susceptibility of offspring to the development of stress-induced obesity.

## 1. Introduction

Obesity is characterized by an excess of adipose tissue and may induce the development of metabolic syndrome. This syndrome is characterized in part by hypertension, dyslipidemia, and type 2 diabetes mellitus [1,2], the progression of which leads to the development of highly lethal diseases such as atherosclerosis. Therefore, it is critical to define the mechanisms underlying the pathogenesis of obesity and to develop preventive strategies. Previous studies in humans have shown that maternal undernutrition during pregnancy and lactation significantly influences the susceptibility of offspring to metabolic syndrome [3,4]. Moreover, in rats and mice, maternal overnutrition has been shown to increase the susceptibility of offspring to the development of obesity, hypertension, and insulin resistance [5,6,7]. These findings have prompted researchers worldwide to study the effects of maternal overnutrition on offspring [5,6,7]; in a recent study in mice, we demonstrated that a maternal high-fat diet during lactation predisposes offspring to the development of diet-induced obesity [8]. Unbalanced maternal nutrition is the first risk factor for the development of metabolic syndrome to which offspring are exposed; therefore, it is important to examine its association with other risk factors for obesity. Apart from excessive energy intake or maternal malnutrition during pregnancy and lactation, risk factors for obesity are ubiquitous in the daily life of humans [1,2]; of these factors, stress has garnered particular attention recently. Stimuli that cause stress in humans include illness, work, family problems, changes in environment, diet, isolation, gender differences, and lack of sleep [9]. These types of stressors, referred to as social stressors, can cause mental illnesses such as depression and can induce bulimia and metabolic abnormalities, thereby increasing the risk of developing metabolic syndrome, obesity, insulin resistance, cardiovascular disease, and fatty liver in humans and mice [10,11,12]. Social stress is a significant risk factor for obesity because it exists for everyone. Mice reared in isolation following weaning experience isolation stress characterized by elevated plasma corticosterone levels, a known stress parameter, compared with rats reared in groups [13]. Moreover, isolation stress has been shown to increase susceptibility to metabolic syndrome in mice [11]. We hypothesized that the interaction between two risk factors for obesity, maternal over nutrition during lactation and isolation stress in offspring, would further increase the susceptibility of offspring to the development of obesity. In the present study, we tested this hypothesis in mice by examining how a maternal high-fat diet during lactation and exposure of offspring to isolation stress influence the susceptibility of offspring to the development of obesity, specifically via the lipid and carbohydrate metabolic pathways. A maternal high-fat diet during lactation was shown to increase the stress sensitivity of offspring, significantly accelerating the progression of obesity. These findings demonstrate that a maternal high-fat diet during lactation and isolation stress experienced by offspring may have combined effects in increasing the susceptibility of offspring to the development of obesity.

## 2. Experimental Section

### 2.1. Animals and Diets

**Figure 1:**
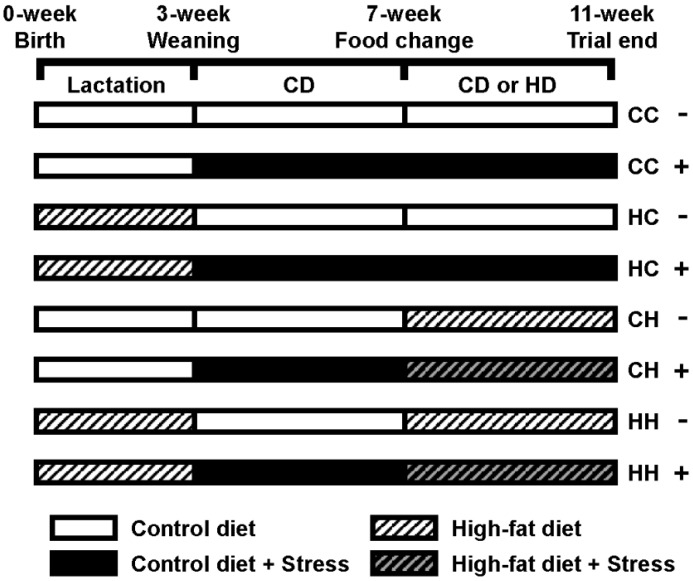
Study protocol. CC− group, dams fed CD during lactation and offspring fed CD and were under ordinary circumstance; CC+ group, dams fed CD during lactation and offspring fed CD and were under isolation stress circumstance; HC− group, dams fed HD during lactation and offspring fed CD and were under ordinary circumstance; HC+ group, dams fed HD during lactation and offspring fed CD and were under isolation stress circumstance; CH− group, dams fed CD during lactation and offspring fed HD and were under ordinary circumstance; CH+ group, dams fed CD during lactation and offspring fed HD and were under isolation stress circumstance; HH−, dams fed HD during lactation and offspring fed HD and were under ordinary circumstance; HH+, dams fed HD during lactation and offspring fed HD and were under isolation stress circumstance.

All procedures were performed in accordance with the Animal Experiment Guidelines of Tohoku University. The animal protocol was approved by the Animal Use Committee at Tohoku University. C57BL/6J mice that were 14–16 days pregnant were obtained from CLEA Japan (Tokyo, Japan). Dams were fed a commercial diet (control diet, CD) (CE-2; CLEA Japan) during pregnancy. There were no significant differences in sex distribution or litter size among the litters. After giving birth, dams were fed a CD or a high-fat diet (HD) (Quick Fat; CLEA Japan) during lactation (3 weeks) (Figure 1). The male offspring were weaned at 3 weeks of age. After weaning, they were fed a CD for 4 weeks (until they were 7 weeks old) and then a CD or an HD for 4 weeks (until they were 11 weeks old). Offspring in the isolation stress treatment were housed at one mouse per cage immediately after weaning; mice were considered to be housed under ordinary circumstances at six mice per cage [11]. Thus, the offspring were assigned to one of eight groups: dams fed a CD during lactation and offspring fed a CD and housed under ordinary circumstances (CC− group, *n =* 6) or isolation stress (CC+ group, *n =* 6), dams fed an HD during lactation and offspring fed a CD and housed under ordinary circumstances (HC− group, *n* = 6) or isolation stress (HC+ group, *n* = 6), dams fed a CD during lactation and offspring fed an HD and housed under ordinary circumstances (CH− group, *n* = 6) or isolation stress (CH+ group, *n* = 6), and dams fed a HD during lactation and offspring fed an HD and housed under ordinary circumstances (HH− group, *n* = 6) or isolation stress (HH+ group, *n* = 6). Mice were housed with free access to food and distilled water in a room at constant temperature and humidity with a 12 h light/12 h dark cycle. The diet composition (CD or HD, g/kg diet) was as follows: nitrogen-free extract, 500 or 465; crude protein, 251 or 242; crude fat, 48 or 136; crude ash, 67 or 52; crude fiber, 42 or 30; and moisture, 93 or 75. The energy content was 343.1 kcal/100 g diet (CD) or 405.5 kcal/100 g diet (HD). The HD used to induce obesity was determined with reference to a previous report [8]. At 11 weeks of age, mice were weighed and then sacrificed by decapitation between 9:00 AM and 11:00 AM. Brain, heart, kidney, liver, lung, spleen, epididymal white adipose tissue, mesenteric white adipose tissue, perinephric white adipose tissue, and serum were collected and stored at −80 °C until assays were performed.

### 2.2. Stress Parameters

Serum corticosterone levels, as a measure of stress, were determined using an ELISA kit (Yanaihara, Fujinomiya, Japan) according to the manufacturer’s protocol. At 11 weeks of age, mice were sacrificed by decapitation between 9:00 AM and 11:00 AM. Then, serum were collected and stored at −80 °C until assays were performed. For each sample, 10 μL of serum was used, and the absorbance was measured using a microplate reader (Infinit F200; Tecan Japan, Kawasaki, Japan) at a wavelength of 450 nm.

### 2.3. Histological Analysis of White Adipose Tissue

Epididymal white adipose tissue was fixed in 10% formalin and embedded in paraffin [14]. Vertical sections (5 μm) were cut, mounted on a glass slide, stained with hematoxylin and eosin, and observed under a microscope (BZ-9000; Keyence, Osaka, Japan). Adipocyte size was determined at 100× magnification using a microscope (BZ-9000). To ensure the accuracy of measurements, we averaged 30 measurements for each animal (10 images of each animal analyzed by three different investigators). Measurements were obtained from six animals per group. Data are presented as the mean ± standard error (SE) for each group.

### 2.4. Biochemical Analyses of Serum

The lipid composition of serum was measured as described previously [14,15]. Triacylglycerol, total cholesterol, phospholipid, non-esterified fatty acid, and glucose levels in serum were measured using commercial enzyme kits (Wako Pure Chemical Industries, Osaka, Japan) according to the manufacturer’s protocol. The insulin level in serum was determined using an ELISA kit (Shibayagi, Shibukawa, Japan).

### 2.5. mRNA Expression Analysis

For real-time quantitative reverse transcription PCR (qRT-PCR), total RNA was isolated from 50 mg of white adipose tissue using an RNeasy Lipid Tissue Mini Kit (Qiagen, Valencia, CA, USA) [14,16], eluted with 30 μL of RNase-free water, and stored at −80 °C until use. The amount of total RNA was determined spectrophotometrically at 260 nm and 280 nm. RNA integrity was confirmed by visualizing intact 28S and 18S ribosomal RNA on a denaturing formaldehyde agarose gel. Using a TP870 Thermal Cycler Dice Real Time System (Takara Bio, Otsu, Japan), mRNA expression levels in white adipose tissue were determined for the following genes: cluster of differentiation 36 (*Cd36*), fatty acid synthase (*Fas*), glucose-6-phosphate dehydrogenase (*G6pdx*), glyceraldehyde-3-phosphate dehydrogenase (*Gapdh*), hormone-sensitive lipase (*Hsl*), lipoprotein lipase (*Lpl*), monocyte chemotactic protein 1 (*Mcp1*), malic enzyme (*Me*), and tumor necrosis factor-α (*Tnfa*). This system allows real-time quantitative detection of PCR products by measuring the increase in fluorescence caused by binding of SYBR Green to double-stranded DNA. In brief, cDNA was prepared from total RNA from white adipose tissue using a Ready-To-Go T-Primed First-Strand Kit (GE Healthcare, Buckinghamshire, UK). The cDNA was subjected to PCR amplification using a SYBR Premix Ex Taq (Perfect Real Time) kit (Takara Bio, Otsu, Japan) and gene-specific primers for *Cd36*, *Fas*, *G6pdx*, *Gapdh*, *Hsl*, *Lpl*, *Mcp1*, *Me*, or *Tnfa*. The primer sequences were as follows: *Cd36* (NM_007643), 5′-ATGGGCTGTGATCGGAACTG-3′ (forward) and 5′-GTCTTCCCAATAAGCATGTCTCC-3′ (reverse); *Fas* (NM_007988), 5′-CCTGGATAGCATTCCGAACCTG-3′ (forward) and 5′-TTCACAGCCTGGGGTCATCTTTGC-3′ (reverse); *G6pdx* (NM_008062), 5′-GAAAGCAGAGTGAGCCCTTC-3′ (forward) and 5′-CATAGGAATTACGGGCAAAGA-3′ (reverse); *Gapdh* (NM_008084), 5′-CATGTTCCAGTATGACTCCACTC-3′ (forward) and 5′-GGCCTCACCCCATTTGATGT-3′ (reverse); *Hsl* (NM_001039507), 5′-TTCTCCAAAGCACCTAGCCAA-3′ (forward) and 5′-TGTGGAAAACTAAGGGCTTGTTG-3′ (reverse); *Lpl* (NM_008509), 5′-GGGAGTTTGGCTCCAGAGTTT-3′ (forward) and 5′-TGTGTCTTCAGGGGTCCTTAG-3′ (reverse); *Mcp1* (NM_011333), 5′-TGTCACCCTTGGAGCTCATG-3′ (forward) and 5′-TTTTTCGACTTTTATCCTCTGTTG-3′ (reverse); *Me* (M29546), 5′-CCTCACCACTCGTGAGGTCAT-3′ (forward) and 5′-CGAAACGCCTCGAATGGT-3′ (reverse); and *Tnfa* (NM_013693), 5′-GCTGTCCCTGCGCTTCA-3′ (forward) and 5′-CTCGTCCCCAATGACATCCT-3′ (reverse). For each gene, the PCR conditions were 95 °C for 10 s and then 40 cycles of 95 °C for 5 s and 60 °C for 31 s. Melting curve analysis was performed after each reaction to confirm that a single reaction product was present. The threshold cycle is the PCR cycle at which an increase in reporter fluorescence above a baseline signal was first detected. The ratio of the *Gapdh* contents of standard and test samples was the normalization factor.

### 2.6. Thiobarbituric Acid Active Substance Assay

To examine oxidative stress caused by aging, we measured the levels of thiobarbituric acid active substances (TBARS) in white adipose tissue as described previously [15].

### 2.7. Statistical Analysis

Results are expressed as mean ± SE. The significance of the effects of high dietary fat intake in dams and isolation stress in offspring, and their interaction, was tested using two-way ANOVAs. When a significant interaction (*p* < 0.05) or a tendency to interaction (*p* < 0.10) was found, individual comparisons were made using Tukey’s test. Differences were considered significant at *p* < 0.05.

## 3. Results

### 3.1. Stress Susceptibility

**Figure 2:**
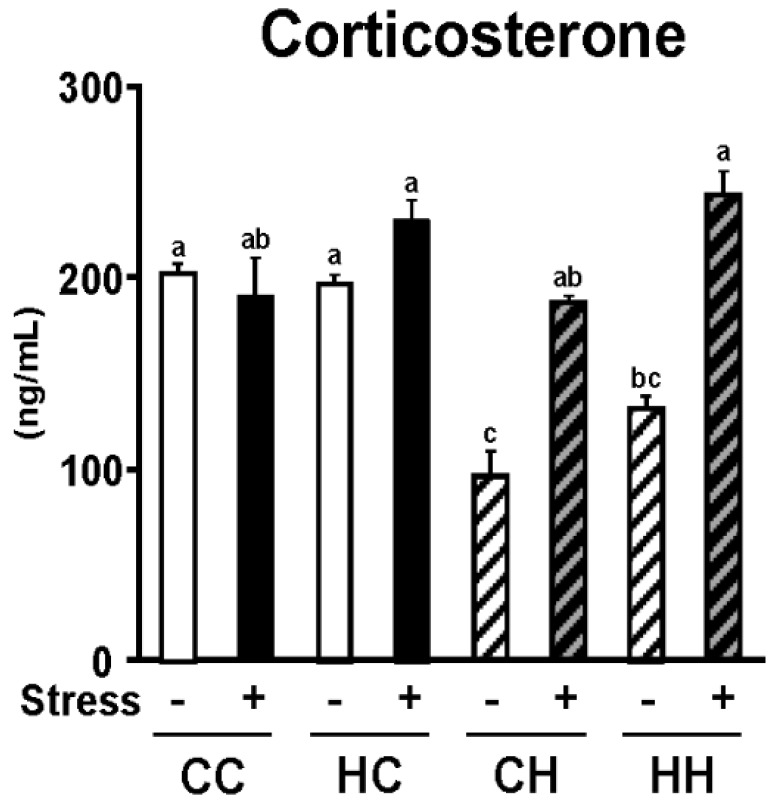
Effects of high dietary fat intake in dams during lactation and isolation stress on serum corticosterone level as a stress parameter. Serum corticosterone level was significantly increased by isolation stress (*p* < 0.01), and also increased by high dietary fat intake in dams during lactation (*p* < 0.01). Moreover, serum corticosterone level had a tendency to increase by a significant interaction between HD intake in dams during lactation and isolation stress of offspring (*p* = 0.097). Values are means ± SE, *n* = 6. Statistical analysis of data was performed by two-way ANOVA followed by the Tukey-Kramer test to identify differences among groups. Different superscript letters indicate significantly different means at *p* < 0.05.

Serum corticosterone level, a known stress parameter, was determined in offspring to examine the effects of a maternal high-fat diet during lactation on stress sensitivity in offspring (Figure 2). Serum corticosterone level was significantly elevated in mice housed one per cage, confirming that these mice were experiencing isolation stress. High-fat feeding of offspring reduced their serum corticosterone level, which was significantly elevated by a maternal high-fat diet. A maternal high-fat diet and exposure of offspring to isolation stress were found to have combined effects in elevating the serum corticosterone level (*p* = 0.097). These findings suggest that a maternal high-fat diet during lactation increases the stress sensitivity of offspring.

### 3.2. Growth Parameters

The effects of a maternal high-fat diet during lactation and exposure of offspring to stress on the growth parameters of offspring were examined (Table 1). Offspring body weight at weaning (three weeks old) was significantly increased by a maternal high-fat diet. The increase in body weight caused by a maternal high-fat diet was no longer observed at seven weeks, following four weeks of a standard diet; however, increases in body weight due to isolation stress were observed. Body weight at the end of the experiment (11 weeks) was significantly increased by isolation stress, but there was no combined effect of a maternal high-fat diet and isolation stress in offspring. Similarly, food intake was significantly increased by isolation stress, but no combined effect of a maternal high-fat diet and isolation stress was observed. In contrast, food intake corrected by weight was significantly increased by a maternal high-fat diet, but there was no combined effect of a maternal high-fat diet and isolation stress. Calorie intake was significantly increased by a maternal high-fat diet alone and in combination with isolation stress. The weights of epididymal and perinephric white adipose tissues were significantly increased by both a maternal high-fat diet and isolation stress as well as by their combination. The weight of mesenteric white adipose tissue was significantly increased by a maternal high-fat diet and isolation stress, but no difference was observed as a result of their combination (*p* = 0.053). These results demonstrate that the weight of white adipose tissue in offspring is increased by isolation stress, and the increase is exacerbated by a maternal high-fat diet during lactation. However, no significant differences were observed in the weight of brain, heart, kidney, liver, lung, or spleen as a result of the combination of a maternal high-fat diet and isolation stress.

**Table 1:** Body weight, food intake and tissue weight in offspring.

	CC	HC	CH	HH	Two-way ANOVA		
Stress	−	−	−	−	Mother	Stress	Interaction
	+	+	+	+			
**Body weight and food intake (g)**							
**3-week**	9.9 ± 1.0	10.9 ± 0.9	9.9 ± 0.9	11.0 ± 0.6	<0.05	ns	ns
	10.4 ± 0.2	11.8 ± 0.3	10.7 ± 0.2	11.7 ± 0.3			
**7-week**	23.3 ± 0.9	24.0 ± 0.9	23.6 ± 0.6	23.5 ± 0.9	ns	0.096	ns
	24.0 ± 0.6	24.4 ± 0.7	23.8 ± 0.3	24.7 ± 0.4			
**11-week**	27.7 ± 1.1	27.6 ± 1.2	30.7 ± 0.6	30.5 ± 1.1	ns	<0.01	ns
	27.9 ± 0.8	28.8 ± 0.8	30.7 ± 1.1	35.3 ± 0.8			
**Food intake (g/day)**							
**Food intake**	3.0 ± 0.2	3.2 ± 0.1	3.0 ± 0.1	3.0 ± 0.1	ns	<0.05	ns
	3.2 ± 0.2	3.3 ± 0.2	3.1 ± 0.1	3.3 ± 0.1			
**Food intake (g/100 g body weight/day)**							
**Food intake**	0.11 ± 0.00	0.12 ± 0.01	0.11 ± 0.00	0.10 ± 0.00	<0.01	ns	ns
	0.11 ± 0.00	0.11 ± 0.00	0.10 ± 0.00	0.09 ± 0.00			
**Calorie intake (calorie/day)**							
**Calorie intake**	10.3 ± 0.0 ^a^	11.1 ± 0.0 ^a^	13.5 ± 0.0 ^d^	12.2 ± 0.0 ^b, c^	<0.01	0.059	<0.01
	11.0 ± 0.4 ^a^	11.3 ± 0.3 ^a^	12.9 ± 0.4 ^c, d^	13.2 ± 0.3 ^c, d^			
**Tissue weight (g/100 g body weight)**							
**Brain**	1.78 ± 0.05	1.81 ± 0.07	1.56 ± 0.03	1.62 ± 0.05	ns	<0.05	ns
	1.72 ± 0.03	1.75 ± 0.04	1.57 ± 0.05	1.43 ± 0.05			
**Heart**	0.53 ± 0.04	0.53 ± 0.03	0.49 ± 0.02	0.47 ± 0.02	ns	ns	ns
	0.52 ± 0.03	0.52 ± 0.03	0.44 ± 0.03	0.42 ± 0.02			
**Kidney**	1.29 ± 0.05	1.17 ± 0.03	1.08 ± 0.02	1.06 ± 0.01	<0.05	0.067	ns
	1.44 ± 0.16	1.23 ± 0.02	1.18 ± 0.04	1.09 ± 0.03			
**Liver**	4.12 ± 0.11	4.15 ± 0.06	3.82 ± 0.01	3.75 ± 0.02	ns	<0.01	ns
	4.40 ± 0.08	4.25 ± 0.06	4.17 ± 0.06	4.08 ± 0.17			
**Lung**	0.73 ± 0.05	0.77 ± 0.08	0.66 ± 0.03	0.72 ± 0.04	ns	ns	ns
	0.69 ± 0.04	0.84 ± 0.06	0.67 ± 0.06	0.66 ± 0.04			
**Spleen**	0.30 ± 0.02	0.30 ± 0.02	0.26 ± 0.01	0.27 ± 0.01	ns	ns	ns
	0.29 ± 0.02	0.26 ± 0.01	0.27 ± 0.02	0.26 ± 0.03			
**White adipose tissue**							
**Epididymal**	0.31 ± 0.04 ^d^	0.24 ± 0.07 ^d^	0.86 ± 0.09 ^b, c^	0.88 ± 0.09 ^b, c^	<0.05	<0.01	<0.01
	0.47 ± 0.06 ^d^	0.53 ± 0.06 ^c, d^	0.93 ± 0.16 ^b^	1.50 ± 0.07 ^a^			
**Mesenteric**	0.11 ± 0.01 ^b^	0.08 ± 0.02 ^e^	0.33 ± 0.04 ^a, b, c^	0.30 ± 0.03 ^b, c, d^	<0.05	<0.01	0.053
	0.19 ± 0.03 ^d, e^	0.19 ± 0.03 ^c, d, e^	0.37 ± 0.05 ^a, b^	0.510.04 ^a^			
**Perinephric**	0.07 ± 0.01 ^e^	0.11 ± 0.02 ^d, e^	0.36 ± 0.04 ^b^	0.36 ± 0.04 ^b^	<0.01	<0.01	<0.05
	0.17 ± 0.04 ^c, d, e^	0.25 ± 0.04 ^b, c, d^	0.37 ± 0.09 ^b, c^	0.67 ± 0.06 ^a^			

Values are means ± SE, *n* = 6. ^a, b, c, d, e^ Different superscript letters indicate significantly different means at *p* < 0.05. ns, no significant difference. CC- group, dams fed CD during lactation and offspring fed CD and were under ordinary circumstance; CC+ group, dams fed CD during lactation and offspring fed CD and were under isolation stress circumstance; HC− group, dams fed HD during lactation and offspring fed CD and were under ordinary circumstance; HC+ group, dams fed HD during lactation and offspring fed CD and were under isolation stress circumstance; CH− group, dams fed CD during lactation and offspring fed HD and were under ordinary circumstance; CH+ group, dams fed CD during lactation and offspring fed HD and were under isolation stress circumstance; HH−, dams fed HD during lactation and offspring fed HD and were under ordinary circumstance; HH+, dams fed HD during lactation and offspring fed HD and were under isolation stress circumstance.

### 3.3. White Adipose Tissue

**Figure 3:**
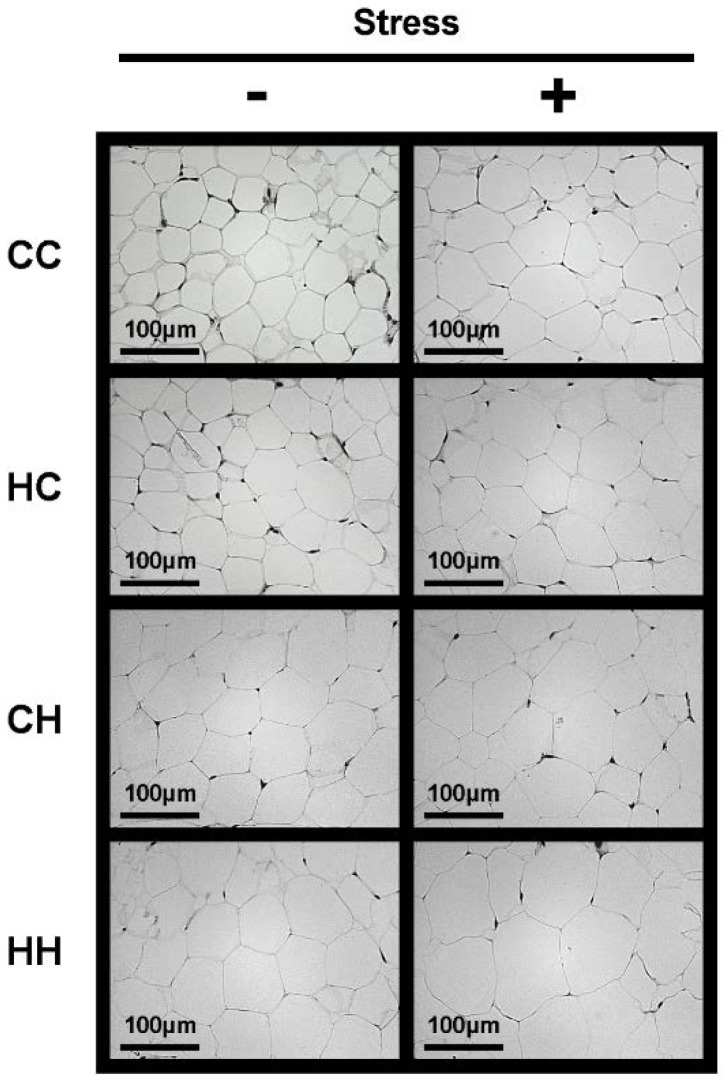
Effects of high dietary fat intake in dams during lactation and isolation stress on adipocytes of offspring. Hematoxylin-eosin staining of white adipose tissue sections from representative mice of each group (scale bar = 100 μm).

**Figure 4:**
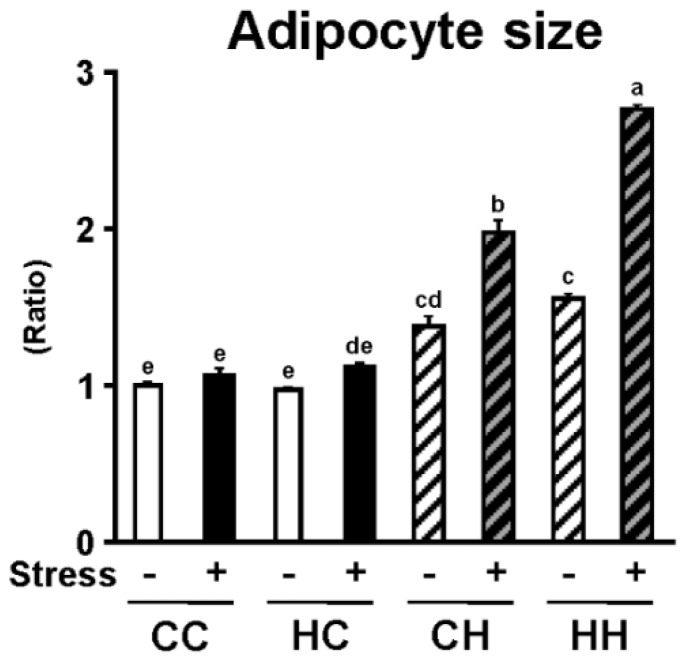
Effects of high dietary fat intake in dams during lactation and isolation stress on adipocyte size of offspring. Adipocyte size was significantly increased by isolation stress (*p* < 0.01), and also increased by high dietary fat intake in dams during lactation (*p* < 0.01). Moreover, adipocyte size was significantly increased by a significant interaction between HD intake in dams during lactation and isolation stress of offspring (*p* < 0.01). Values are means ± SE, *n* = 6. Statistical analysis of data was performed by two-way ANOVA followed by the Tukey-Kramer test to identify differences among groups. Different superscript letters indicate significantly different means at *p* < 0.05.

Following the observation that the weight of white adipose tissue was significantly increased by the combination of a maternal high-fat diet and isolation stress in offspring, the size of white adipocytes, which are strongly involved in the progression of obesity, was examined. White adipose tissue sections stained with hematoxylin and eosin were examined under a microscope to determine adipocyte size (Figure 3). Adipocyte hypertrophy was observed following a maternal high-fat diet and isolation stress in offspring. The area of adipocytes was then calculated and compared among treatment groups (Figure 4). Significant adipocyte hypertrophy was induced by a maternal high-fat diet and isolation stress and exacerbated by their combination. These observations demonstrate that a maternal high-fat diet and isolation stress promote visceral fat accumulation and adipocyte hypertrophy, accelerating the progression of obesity through their combined effects.

### 3.4. Expression of mRNA for Genes Related to Lipid Metabolism and Inflammation in White Adipose Tissue

The expression of mRNA for genes involved in lipid metabolism and inflammation in white adipose tissue was analyzed by qRT-PCR to examine the mechanism by which the combination of a maternal high-fat diet and isolation stress in offspring promotes visceral fat accumulation and adipocyte hypertrophy (Table 2). *Fas* and *Me* are both involved in fatty acid biosynthesis. The expression of *Fas* mRNA was significantly increased by isolation stress alone and in combination with a maternal high-fat diet. The expression of *Me* mRNA was significantly increased by a maternal high-fat diet, but no difference was observed as a result of the combination of a maternal high-fat diet with isolation stress. The expression of mRNA for three genes (*Lpl*, *Hsl*, and *Cd36*) involved in lipid influx from blood into adipocytes was also determined. The expression of mRNA for *Lpl*, which is involved in lipoprotein degradation, increased as a result of a maternal high-fat diet (*p* = 0.064), and the increase was significantly greater in combination with isolation stress. The expression of mRNA for *Hsl*, which is involved in fatty acid secretion from adipocytes into blood, was significantly reduced by isolation stress, but no difference was observed as a result of the combination of isolation stress with a maternal high-fat diet. No significant difference was observed in the expression of mRNA for *Cd36*, which is involved in fatty acid influx. These findings suggest that a maternal high-fat diet and isolation stress in offspring have combined effects in promoting fatty acid synthesis and lipid influx in white adipose tissue, thereby accelerating adipocyte hypertrophy and the increase in white adipose tissue weight in offspring. The expression of mRNA for two genes involved in inflammation, *Mcp1* and *Tnfa*, was also determined. The expression of *Mcp1* mRNA was significantly increased by a maternal high-fat diet and isolation stress, but no difference was observed as a result of their combination. The expression of *Tnfa* mRNA was significantly increased by a maternal high-fat diet, but no difference was observed as a result of the combination of a maternal high-fat diet with isolation stress. These observations suggest that a maternal high-fat diet significantly affects the expression of mRNA for genes related to inflammation, although no combined effect with isolation stress was observed.

**Table 2:** mRNA expression levels for lipid and carbohydrate metabolism and inflammation-related genes in white adipose tissue of offspring.

	CC	HC	CH	HH	Two-way ANOVA		
Stress	−	−	−	−	Mother	Stress	Interaction
	+	+	+	+			
**(Ratio)**							
**Cd36**	1.00 ± 0.28	0.94 ± 0.40	1.15 ± 0.43	1.89 ± 0.43	ns	ns	ns
	1.37 ± 0.21	1.32 ± 0.71	0.24 ± 0.03	0.81 ± 0.23			
**Fas**	1.00 ± 0.21 ^c, d^	0.47 ± 0.13 ^d^	3.47 ± 1.05 ^a, b, c^	2.64 ± 0.36 ^b, c, d^	ns	<0.05	<0.05
	0.57 ± 0.13 ^c, d^	1.28 ± 0.28 ^b, c, d^	4.01 ± 1.19 ^a, b^	5.77 ± 0.63 ^a^			
**Hsl**	1.00 ± 0.44	1.09 ± 0.38	0.24 ± 0.13	0.32 ± 0.12	ns	< 0.05	ns
	0.59 ± 0.09	0.27 ± 0.16	0.11 ± 0.01	0.09 ± 0.02			
**Lpl**	1.00 ± 0.14 ^a, b^	0.38 ± 0.11 ^b^	1.04 ± 0.13 ^a, b^	1.39 ± 0.19 ^a, b^	0.064	ns	<0.05
	0.51 ± 0.10 ^a, b^	1.28 ± 0.49 ^a, b^	0.81 ± 0.06 ^a, b^	1.43 ± 0.11 ^a^			
**Me**	1.00 ± 0.23	0.63 ± 0.11	0.92 ± 0.07	2.92 ± 0.70	<0.05	ns	ns
	1.51 ± 0.25	1.28 ± 0.47	0.78 ± 0.19	2.35 ± 0.25			
**MCP-1**	1.00 ± 0.23	1.28 ± 0.40	1.97 ± 0.35	3.47 ± 0.83	<0.01	<0.05	ns
	1.16 ± 0.12	2.00 ± 0.61	4.22 ± 0.76	4.68 ± 1.09			
**TNF-α**	1.00 ± 0.43	1.34 ± 0.30	4.09 ± 1.40	4.51 ± 1.77	<0.01	ns	ns
	2.19 ± 0.57	2.33 ± 0.52	4.29 ± 0.80	6.40 ± 1.39			

Values are means ± SE, *n* = 6. ^a, b, c, d^ Different superscript letters indicate significantly different means at *p* < 0.05. ns, no significant difference. Cd36, cluster of differentiation 36; Fas, fatty acid synthase; Hsl, hormone sensitive lipase; Lpl, lipoprotein lipase; Me, malic enzyme. CC− group, dams fed CD during lactation and offspring fed CD and were under ordinary circumstance; CC+ group, dams fed CD during lactation and offspring fed CD and were under isolation stress circumstance; HC− group, dams fed HD during lactation and offspring fed CD and were under ordinary circumstance; HC+ group, dams fed HD during lactation and offspring fed CD and were under isolation stress circumstance; CH− group, dams fed CD during lactation and offspring fed HD and were under ordinary circumstance; CH+ group, dams fed CD during lactation and offspring fed HD and were under isolation stress circumstance; HH−, dams fed HD during lactation and offspring fed HD and were under ordinary circumstance; HH+, dams fed HD during lactation and offspring fed HD and were under isolation stress circumstance.

### 3.5. Lipid and Carbohydrate Parameters in Serum

Parameters of lipid and carbohydrate metabolism in offspring were determined (Table 3). Serum triacylglycerol levels were significantly reduced as a result of a maternal high-fat diet and isolation stress, and serum free fatty acid levels were significantly reduced as a result of isolation stress. However, there were no combined effects of a maternal high-fat diet and isolation stress on either of these parameters. No significant change was observed in serum total cholesterol levels. Serum phospholipid levels were significantly elevated as a result of a maternal high-fat diet, and they increased further as a result of the combination of a maternal high-fat diet with isolation stress (*p* = 0.083). These results indicate that both a maternal high-fat diet and isolation stress in offspring influence lipid parameters, but no combined effects were observed. Isolation stress significantly elevated serum glucose levels, which appeared to cause an increase in serum insulin levels as well (*p* = 0.064). These observations suggest that isolation stress in offspring significantly affects carbohydrate parameters, although no combined effect with a maternal high-fat diet was observed. Serum adiponectin levels were significantly elevated as a result of isolation stress, and serum leptin levels were significantly elevated as a result of a maternal high-fat diet and isolation stress. In addition, TBARS levels in white adipose tissue were significantly elevated as a result of isolation stress. These observations suggest that isolation stress in offspring significantly affects adiponectin, leptin, and TBARS levels, although no combined effect with a maternal high-fat diet was observed.

**Table 3:** Serum lipid and carbohydrate metabolism parameters and stress parameter, and TBARS in white adipose tissue (WAT) of offspring.

	CC	HC	CH	HH	Two-way ANOVA		
Stress	−	−	−	−	Mother	Stress	Interaction
	+	+	+	+			
**TG (mmol/L)**	1.08 ± 0.02	0.94 ± 0.07	1.44 ± 0.08	1.22 ± 0.08	<0.05	<0.01	ns
	0.90 ± 0.07	0.73 ± 0.08	1.09 ± 0.05	1.20 ± 0.08			
**TC (mmol/L)**	1.54 ± 0.09	1.70 ± 0.12	2.19 ± 0.07	2.24 ± 0.03	ns	ns	ns
	1.66 ± 0.04	1.61 ± 0.08	2.43 ± 0.15	2.56 ± 0.17			
**PL (mmol/L)**	2.27 ± 0.13 ^d^	2.65 ± 0.07 ^b, c^	2.69 ± 0.05 ^a, b, c^	2.95 ± 0.03 ^a^	<0.01	ns	0.083
	2.19 ± 0.04 ^d^	2.00 ± 0.08 ^c, d^	2.98 ± 0.05 ^a, b, c^	3.01 ± 0.10 ^a^			
**NEFA (mEq/L)**	0.91 ± 0.06	0.91 ± 0.08	0.85 ± 0.01	0.79 ± 0.02		<0.01	ns
	0.76 ± 0.06	0.67 ± 0.01	0.80 ± 0.03	0.77 ± 0.05			
**Glucose (mmol/L)**	8.4 ± 0.7	8.7 ± 0.4	9.9 ± 0.6	10.8 ± 0.4	0.100	<0.01	ns
	12.6 ± 0.9	12.8 ± 0.8	14.1 ± 0.8	16.4 ± 1.3			
**Insulin (ng/mL)**	0.43 ± 0.16	1.54 ± 0.87	0.46 ± 0.11	1.56 ± 0.84	ns	0.064	ns
	1.88 ± 0.94	2.18 ± 1.00	2.82 ± 1.14	2.03 ± 1.32			
**Adiponectin (μg/mL)**	3.19 ± 0.62	3.89 ± 0.26	3.51 ± 0.38	3.88 ± 0.27	ns	<0.05	ns
	2.56 ± 0.12	2.86 ± 0.45	2.43 ± 0.26	3.57 ± 0.59			
**Leptin (ng/mL)**	0.29 ± 0.01	0.34 ± 0.01	0.74 ± 0.01	0.81 ± 0.04	<0.01	<0.01	ns
	0.37 ± 0.02	0.47 ± 0.02	0.92 ± 0.03	1.10 ± 0.13			
**WAT TBARS (nmol/g WAT)**	2.05 ± 0.19	2.82 ± 0.36	2.71 ± 0.21	2.91 ± 0.15	ns	<0.05	ns
	3.01 ± 0.29	3.02 ± 0.28	3.12 ± 0.15	3.39 ± 0.41			

Values are means ± SE, *n* = 6. ^a, b, c, d^ Different superscript letters indicate significantly different means at *P*<0.05. ns, no significant difference. TG, triacylglycerol; TC, total cholesterol; PL, phospholipid; NEFA, non-esterified fatty acid. CC− group, dams fed CD during lactation and offspring fed CD and were under ordinary circumstance; CC+ group, dams fed CD during lactation and offspring fed CD and were under isolation stress circumstance; HC- group, dams fed HD during lactation and offspring fed CD and were under ordinary circumstance; HC+ group, dams fed HD during lactation and offspring fed CD and were under isolation stress circumstance; CH− group, dams fed CD during lactation and offspring fed HD and were under ordinary circumstance; CH+ group, dams fed CD during lactation and offspring fed HD and were under isolation stress circumstance; HH−, dams fed HD during lactation and offspring fed HD and were under ordinary circumstance; HH+, dams fed HD during lactation and offspring fed HD and were under isolation stress circumstance.

## 4. Discussion

We recently demonstrated that maternal overnutrition during lactation increases the susceptibility of offspring to the development of diet-induced obesity [8]. It has also been reported that social stress, such as isolation, can be a risk factor for the development of obesity [10]. The present study, in mice, examined the combined effects of a maternal high-fat diet during lactation and exposure of offspring to isolation stress on the susceptibility of offspring to the development of obesity. Serum corticosterone levels were determined to assess the effects of a maternal high-fat diet during lactation on stress sensitivity in offspring. Serum corticosterone levels were significantly elevated by isolation stress and a maternal high-fat diet, and the two treatments appeared to have combined effects in elevating serum corticosterone levels (Figure 2). Several studies have demonstrated that serum corticosterone levels increase with exposure to chronic isolation stress or intense stress; serum corticosterone is thus known to be an important stress parameter [17,18]. In addition, serum corticosterone has been shown to play a critical role in the progression of obesity because it elevates blood glucose levels [19]. Thus, alteration of serum corticosterone levels is a critical parameter of both stress exposure and the development of obesity. We have recently demonstrated that a maternal high-fat diet during lactation increases the lipid and calorie contents of dam’s milk [8]. Therefore, we hypothesized that if offspring of a mother that consumed a high-fat diet were fed a high-calorie diet during infancy, their steady-state blood glucose level would be elevated, which is known to alleviate stress. Upon exposure to stress, blood glucose levels in these offspring would increase more than those in offspring fed a normal diet during infancy, leading to secretion of larger amounts of corticosterone. In addition, serum corticosterone levels are known to be reduced by consumption of a high-fat diet [20]. In the present study, high-fat feeding of offspring reduced their corticosterone levels (Figure 2). However, corticosterone levels were found to be high in the HH+ group, in which visceral fat accumulation was strongly promoted, suggesting that individuals in the HH+ group experienced greater stress. These findings suggest that a maternal high-fat diet during lactation increases the stress sensitivity of offspring, thereby facilitating the development of stress-induced obesity. Moreover, significant adipocyte hypertrophy was observed as a result of the combined effects of a maternal high-fat diet and isolation stress (Figure 3 and Figure 4). This demonstrates that a maternal high-fat diet during lactation and isolation stress have combined effects in facilitating the development of obesity in offspring.

The mechanisms underlying adipocyte hypertrophy were examined next. The mRNA expression levels of Lpl, which facilitates lipid influx from blood [21], and Fas, which facilitates fatty acid synthesis [22], were increased by the combined effects of a maternal high-fat diet and isolation stress in offspring (Table 2). This suggests that triacylglycerol accumulation in white adipose tissue was facilitated by increased fatty acid synthesis and lipid influx from blood. The expression of *Fas* and *Lpl* mRNA in adipose tissue was increased by a maternal high-fat diet during lactation, consistent with our previous report [8]. Social stress has also been reported to increase expression of *Fas* [23]. The present study demonstrated that the expression of these genes in adipose tissue is further increased by the combination of a maternal high-fat diet during lactation and isolation stress. In addition, in this study, an increase in calorie intake was observed as a result of the combination of a maternal high-fat diet and isolation stress in offspring. Therefore, calorie intake may also be related to adipocyte hypertrophy.

Offspring body weight at weaning was significantly increased by a maternal high-fat diet, consistent with our previous report [8] (Table 1). The increase in food intake observed as a result of isolation stress was also consistent with a previous report [10] (Table 1), suggesting its involvement in the progression of obesity. A maternal high-fat diet significantly reduced kidney weight, whereas isolation stress significantly reduced brain weight and increased liver weight (Table 1). These changes occurred as a result of the body weight increase associated with visceral fat accumulation, and no significant changes were observed when the values were not corrected for body weight.

Serum glucose level, which is known to increase in response to stress [9], was significantly increased as a result of exposure of offspring to isolation stress (Table 3). Serum insulin level appeared to increase as a result of isolation stress (Table 3), which may be a response to the increased blood glucose level caused by isolation stress. Moreover, insulin, as well as corticosterone, has been reported to facilitate lipid synthesis in white adipose tissue [24]. This suggests that the observed decrease in serum triacylglycerol and free fatty acid levels was a result of these molecules being used for lipid synthesis (Table 3). Additionally, the significant decrease in the mRNA expression level of Hsl, which functions in triacylglycerol degradation, may also have played a role (Table 2). These findings demonstrate that several parameters of serum lipid and carbohydrate metabolism are significantly influenced by a maternal high-fat diet and isolation stress. In addition, it has been reported that adipocyte hypertrophy causes an increase in serum leptin level and a decrease in serum adiponectin level [14,15]. Similar observations were made in this study. These findings demonstrate that serum leptin and adiponectin levels are significantly influenced by a maternal high-fat diet and isolation stress. It has also been shown that adipocyte hypertrophy causes an increase in mRNA expression levels for inflammation-related genes such as *Mcp1* and *Tnfa* [25]. Similar phenomena were observed in this study. These findings demonstrate that mRNA expression levels for inflammation-related genes are significantly influenced by a maternal high-fat diet and isolation stress. Moreover, our study confirms previous reports that the inflammatory condition increases oxidative stress [8,16], demonstrating that the level of oxidative stress is significantly influenced by a maternal high-fat diet and isolation stress.

In this study, corticosterone level was the only stress parameter used. Although other studies have used corticosterone level as the only stress parameter, it will be necessary to confirm the degree of stress using several stress parameters in the future.

The weaning weight of the offspring (three weeks old) was increased by a maternal high-fat diet during lactation. This difference disappeared after offspring were fed a control diet from three to seven weeks of age. However, the risk of offspring becoming obese at 11 weeks of age was changed by the diet of the dams. This suggests that metabolic memory during infancy influences future metabolism in offspring. Recent studies suggest that epigenetic mechanisms such as DNA methylation and histone acetylation are involved in this phenomenon. For example, it is known that DNA methylation influences the mRNA level of genes related to lipid metabolism and that high dietary fat intake alters DNA methylation [26]. Furthermore, maternal nutritional status during pregnancy and lactation can influence the methylation of genes related to lipid metabolism in offspring [27,28]. Therefore, these epigenetic mechanisms may underlie the results obtained in this study. Further studies are needed to confirm this hypothesis. Fatty acid biosynthesis may also be involved in the observed changes in white adipose tissue weights because the mRNA level of Fas, which promotes fatty acid biosynthesis [22], was significantly increased by high-fat feeding of offspring. This increase may account for the increased lipid accumulation in adipocytes. Epigenetic mechanisms may also underlie this result, and further studies are needed to examine this hypothesis as well.

In humans, maternal overnutrition has been shown to increase the susceptibility of offspring to the development of obesity, hypertension, and insulin resistance [5,6,7]. In addition, social stressors (events in everyday life, including illness, work, family problems, changes in environment, diet, isolation, gender differences, and lack of sleep) have been shown to cause mental illnesses such as depression and to induce bulimia and metabolic abnormalities, thereby increasing the risk of developing metabolic syndrome, obesity, insulin resistance, cardiovascular disease, and fatty liver [9,10,11,12]. Therefore, the possibility that a similar phenomenon occurs in humans was suggested, though the present study was an examination of mice.

## 5. Conclusions

Maternal nutritional status is the first risk factor for the development of obesity to which offspring are exposed; therefore, it is important to elucidate its association with other risk factors for the development of obesity. The findings of the present study demonstrate that a maternal high-fat diet during lactation and isolation stress in offspring may interact to significantly facilitate the development of obesity.

## References

[1] Haslam D.W., James W.P. (2005). Obesity. Lancet.

[2] McMillen I.C., Rattanatray L., Duffield J.A., Morrison J.L., MacLaughlin S.M., Gentili S., Muhlhausler B.S. (2009). The early origins of later obesity: Pathways and mechanisms. Adv. Exp. Med. Biol..

[3] Barker D.J., Hales C.N., Fall C.H., Osmond C., Phipps K., Clark P.M. (1993). Type 2 (non-insulin-dependent) diabetes mellitus, hypertension and hyperlipidaemia (syndrome X): Relation to reduced fetal growth. Diabetologia.

[4] Barker D.J., Osmond C. (1986). Infant mortality, childhood nutrition, and ischaemic heart disease in England and Wales. Lancet.

[5] Khan I.Y., Dekou V., Douglas G., Jensen R., Hanson M.A., Poston L., Taylor P.D. (2005). A high-fat diet during rat pregnancy or suckling induces cardiovascular dysfunction in adult offspring. Am. J. Physiol. Regul. Integr. Comp. Physiol..

[6] Elahi M.M., Cagampang F.R., Mukhtar D., Anthony F.W., Ohri S.K., Hanson M.A. (2009). Long-term maternal high-fat feeding from weaning through pregnancy and lactation predisposes offspring to hypertension, raised plasma lipids and fatty liver in mice. Br. J. Nutr..

[7] Howie G.J., Sloboda D.M., Kamal T., Vickers M.H. (2009). Maternal nutritional history predicts obesity in adult offspring independent of postnatal diet. J. Physiol..

[8] Tsuduki T., Kitano Y., Honma T., Kijima R., Ikeda I. (2013). High dietary fat intake during lactation promotes development of diet-induced obesity in male offspring of mice. J. Nutr. Sci. Vitaminol..

[9] Moles A., Bartolomucci A., Garbugino L., Conti R., Caprioli A., Coccurello R., Rizzi R., Ciani B., D’Amato F.R. (2006). Psychosocial stress affects energy balance in mice: Modulation by social status. Psychoneuroendocrinology.

[10] Kuo L.E., Kitlinska J.B., Tilan J.U., Li L., Baker S.B., Johnson M.D., Lee E.W., Burnett M.S., Fricke S.T., Kvetnansky R. (2007). Neuropeptide Y acts directly in the periphery on fat tissue and mediates stress-induced obesity and metabolic syndrome. Nat. Med..

[11] Sakakibara H., Suzuki A., Kobayashi A., Motoyama K., Matsui A., Sayama K., Kato A., Ohashi N., Akimoto M., Nakayama T., Shimoi K. (2012). Social isolation stress induces hepatic hypertrophy in C57BL/6J mice. J. Toxicol. Sci..

[12] Wolkowitz O.M., Reus V.I., Mellon S.H. (2011). Of sound mind and body: Depression, disease, and accelerated aging. Dialogues Clin. Neurosci..

[13] Toth M., Mikics E., Tulogdi A., Aliczki M., Haller J. (2011). Post-weaning social isolation induces abnormal forms of aggression in conjunction with increased glucocorticoid and autonomic stress responses. Horm. Behav..

[14] Takasaki M., Honma T., Yanaka M., Sato K., Shinohara N., Tanaka Y., Tsuduki T., Ikeda I. (2012). Continuous intake of a high-fat diet beyond one generation promotes lipid accumulation in liver and white adipose tissue of female mice. J. Nutr. Biochem..

[15] Tsuduki T., Kikuchi I., Kimura T., Nakagawa K., Miyazawa T. (2013). Intake of mulberry 1-deoxynojirimycin prevents diet-induced obesity through increases in adiponectin in mice. Food Chem..

[16] E S., Kijima R., Honma T., Yamamoto K., Hatakeyama Y., Kitano Y., Kimura T., Nakagawa K., Miyazawa T., Tsuduki T. (2014). 1-Deoxynojirimycin attenuates high glucose-accelerated senescence in human umbilical vein endothelial cells. Exp. Gerontol..

[17] Bartolomucci A., Palanza P., Sacerdote P., Panerai A.E., Sgoifo A., Dantzer R., Parmigiani S. (2005). Social factors and individual vulnerability to chronic stress exposure. Neurosci. Biobehav. Rev..

[18] Finger B.C., Dinan T.G., Cryan J.F. (2011). High-fat diet selectively protects against the effects of chronic social stress in the mouse. Neuroscience.

[19] Seckl J.R., Morton N.M., Chapman K.E., Walker B.R. (2004). Glucocorticoids and 11 β-hydroxysteroid dehydrogenase in adipose tissue. Recent. Prog. Horm. Res..

[20] Paternain L., García-Diaz D.F., Milagro F.I., González-Muniesa P., Martinez J.A., Campión J. (2011). Regulation by chronic-mild stress of glucocorticoids, monocyte chemoattractant protein-1 and adiposity in rats fed on a high-fat diet. Physiol. Behav..

[21] Wang H., Eckel R.H. (2009). Lipoprotein lipase: From gene to obesity. Am. J. Physiol. Endocrinol. Metab..

[22] Semenkovich C.F. (1997). Regulation of fatty acid synthase (FAS). Prog. Lipid Res..

[23] Motoyama K., Nakai Y., Miyashita T., Fukui Y., Morita M., Sanmiya K., Sakakibara H., Matsumoto I., Abe K., Yakabe T. (2009). Isolation stress for 30 days alters hepatic gene expression profiles, especially with reference to lipid metabolism in mice. Physiol. Genomics.

[24] Peckett A.J., Wright D.C., Riddell M.C. (2011). The effects of glucocorticoids on adipose tissue lipid metabolism. Metabolism.

[25] Wooa S.J., Limb K., Parkc S.Y., Jungc M.Y., Limd H.S., Jeond M.-G., Leed S.-I., Parka B.-H. (2015). Endogenous conversion of *n*-6 to *n*-3 polyunsaturated fatty acids attenuates K/BxN serum-transfer arthritis in fat-1 mice. J. Nutr. Biochem..

[26] Fujiki K., Kano F., Shiota K., Murata M. (2009). Expression of the peroxisome proliferator activated receptor gamma gene is repressed by DNA methylation in visceral adipose tissue of mouse models of diabetes. BMC Biol..

[27] Jousse C., Parry L., Lambert-Langlais S., Maurin A.C., Averous J., Bruhat A., Carraro V., Tost J., Letteron P., Chen P. (2011). Perinatal undernutrition affects the methylation and expression of the leptin gene in adults: Implication for the understanding of metabolic syndrome. FASEB J..

[28] Aagaard-Tillery K.M., Grove K., Bishop J., Ke X., Fu Q., McKnight R., Lane R.H. (2008). Developmental origins of disease and determinants of chromatin structure: Maternal diet modifies the primate fetal epigenome. J. Mol. Endocrinol..

